# Interprofessional advanced access – a quality improvement protocol for expanding access to primary care services

**DOI:** 10.1186/s12913-021-06839-w

**Published:** 2021-08-13

**Authors:** Isabelle Gaboury, Mylaine Breton, Kathy Perreault, François Bordeleau, Sarah Descôteaux, Lara Maillet, Catherine Hudon, Yves Couturier, Arnaud Duhoux, Brigitte Vachon, Benoit Cossette, Isabel Rodrigues, Marie-Eve Poitras, Christine Loignon, Helen-Maria Vasiliadis

**Affiliations:** 1 Department of Family Medicine and Emergency Medicine, Faculty of Medicine and Health Sciences, Université de Sherbrooke, Sherbrooke, Canada; 2 Department of community health sciences, Faculty of Medicine and Health Sciences, Université de Sherbrooke, Sherbrooke, Canada; 3 GMF-U Saint-Jean-sur-Richelieu, Saint-Jean-sur-Richelieu, Canada; 4 École Nationale d’Administration Publique, Montreal, Canada; 5 School of social work, Faculty of letters and social sciences, Université de Sherbrooke, Sherbrooke, Canada; 6 Faculty of Nursing, Université de Montréal, Montreal, Canada; 7 School of Rehabilitation, Faculty of Medicine, Université de Montréal, Montreal, Canada; 8 Department of Family Medicine and Emergency Medicine, Faculty of Medicine, Université de Montréal, Montreal, Canada

**Keywords:** Advanced Access, Primary care, Quality improvement, Practice facilitation, Interprofessional collaboration, Organizational change

## Abstract

**Background:**

The Advanced Access (AA) Model has shown considerable success in improving timely access for patients in primary care settings. As a result, a majority of family physicians have implemented AA in their organizations over the last decade. However, despite its widespread use, few professionals other than physicians and nurse practitioners have implemented the model. Among those who have integrated it to their practice, a wide variation in the level of implementation is observed, suggesting a need to support primary care teams in continuous improvement with AA implementation. This quality improvement research project aims to document and measure the processes and effects of practice facilitation, to implement and improve AA within interprofessional teams.

**Methods:**

Five primary care teams at various levels of organizational AA implementation will take part in a quality improvement process. These teams will be followed independently over PDSA (Plan-Do-Study-Act) cycles for 18 months. Each team is responsible for setting their own objectives for improvement with respect to AA.

The evaluation process consists of a mixed-methods plan, including semi-structured interviews with key members of the clinical and management teams, patient experience survey and AA-related metrics monitored from Electronic Medical Records over time.

**Discussion:**

Most theories on organizational change indicate that practice facilitation should enable involvement of stakeholders in the process of change and enable improved interprofessional collaboration through a team-based approach. Improving access to primary care services is one of the top priorities of the Quebec’s ministry of health and social services. This study will identify key barriers to quality improvement initiatives within primary care and help to develop successful strategies to help teams improve and broaden implementation of AA to other primary care professionals.

**Supplementary Information:**

The online version contains supplementary material available at 10.1186/s12913-021-06839-w.

## Background

### Advanced Access Model

**Fig. 1:**
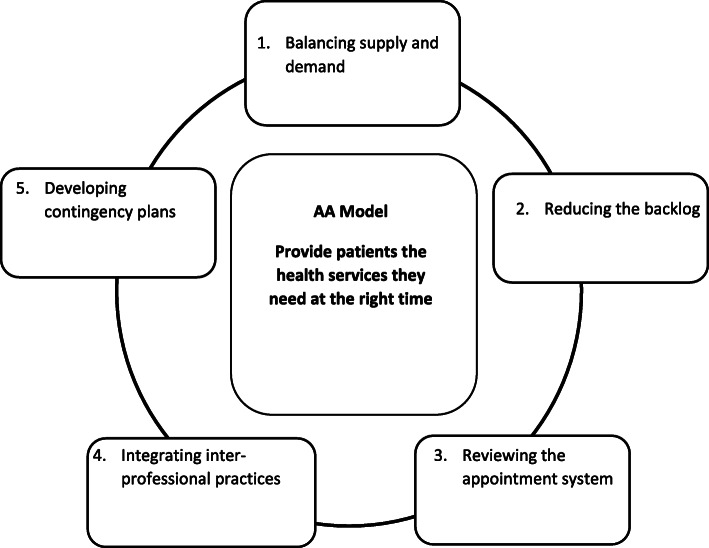
The AA model

Various organisational innovations have been developed in primary care to improve access, such as the Advanced access (AA) model [1]. Initially developed in the United States in response to a perceived lack of access to healthcare services, it has since also been implemented in the United Kingdom and Canada, with many studies conducted over the years to demonstrate its effectiveness [2–6]. AA aims to improve access for patients through a tailored approach, so that they can access the services they need from the right professional at the right time [7]. As a model, it is based on five main principles (Fig. 1); (1) Balancing supply and demand, by reviewing the patients’ requests for appointments according to their level of urgency and adjusting the number of available appointments as needed; (2) Reducing the backlog, by eliminating waiting lists to adjust supply and anticipate demand in a more timely matter; (3) Reviewing the appointment system to focus on short-term planning of supply and maintaining availability for urgent cases; (4) Integrating inter-professional practice with increased collaboration between healthcare professionals so that patients can access the most appropriate services as needed; (5) Developing contingency plans in order to better prepare for predictable variations in supply or demand, such as flu seasons, staff vacations or healthcare professional absences [1].

To date, AA has mostly been adopted by physicians, and to a certain extent by nurse practitioners [8, 9]. Examples of its adoption by other professionals working in primary care settings, such as pharmacists, psychologists or social workers, are not documented in the literature; although previous research experiences among our team have shown that many primary healthcare practitioners could benefit from this approach, suggesting a need to explore the possibilities and adaptations needed to transpose the principles of AA to a wider range of professionals.

Implementing advanced access requires major organizational change, which implies reorganizing the practice of all team members (administrative staff and health professionals) to be more patient-focused and efficient [10, 11]. To this end, the Model for Improvement, which focuses on developing a strong understanding of the system as a whole to help foster structural and organizational changes, is especially well-suited [11].

### Quality improvement (QI)

Promoting organizational change, such as the implementation of AA in primary care, is a constant struggle for healthcare systems. One of the most widely known models used by teams aiming to improve care for patients is the Model for Improvement [12]. As an approach, it is designed to promote, structure and sustain changes in organizations in order to improve both processes and outcomes [13]. It aims to build knowledge about specific organizations, to focus on improvement of the system as a whole and to increase healthcare quality and outcomes [14]. Using three basic questions, the Model for Improvement engages stakeholders in a reflective practice process to define clear goals (What are we trying to accomplish?); identify measures to assess progress towards these goals (How will we know that a change is an improvement?); and develop changes to achieve the goals (What change can we make that will result in improvement?)[12].

These changes are then implemented through iterative implementation cycles [15–17] of four stages (recognized through the acronym PDSA) where a change is: (1) planned based on evidence from data, community feedback and/or stakeholder experience (Plan); (2) carried out while documenting its effects (Do); (3) analyzed by measuring the results achieved, comparing them to expected results and appreciating the impact of change (Study); and finally (4) refined, either by maintaining or adjusting actions in future cycles or by expanding its scale (Act) [12].

While other approaches promote organizational change [12], they are often based on improving specific processes, either by eliminating waste from the system (e.g. LEAN) [18] or reducing variability and errors in the process (e.g. Six-Sigma) [19]. The Model for Improvement applies a broader scope; rather than focusing on the process itself as the main object of change, it aims at a better understanding of the system itself in order to address more general objectives, such as cultural or structural changes within the organization or, in the case of this study, implementing a new model and processes altogether [14].

### Facilitation to support QI

While QI interventions have been shown to be a strong approach to implement change in various settings [20–22], multiple strategies are used to foster organizational changes [23, 24]. To support the implementation of QI interventions, experts internal or external to the organization can accompany the process. This is called practice facilitation. Practice facilitation is one of the proven ways to foster organizational change and instill a continuous quality improvement culture in primary care settings [25, 26]. Practice facilitation is especially useful as a stand-alone intervention, compared with more limited strategies such as academic detailing, or audit and feedback [27]. Although practice facilitation can also be used effectively in combination with other techniques [27, 28], studies have shown that tailoring facilitation interventions to the individual is a determining factor to achieve buy-in from stakeholders and, more generally, to implement changes in local settings successfully [22, 29–31]. Using external change agents in the practice facilitation model also appears to strongly facilitate organizational change, especially for smaller settings and primary care services [31–34]. Other enablers of practice facilitation included sustained interactions between facilitators and practices, frequency and timeframe of ongoing practice facilitation, funding, and patient and partner engagement [31–34].

### Context of intervention

AA has been recognized by Quebec’s Ministry of Health and Social Services (MSSS) as one of the key factors for successful patient access to healthcare, in response to a growing concern about the lack of timely access to family physicians in the province [35, 36]. Since 2012, AA has been heavily promoted in primary care settings across the province [7]. This led to a huge organizational change within primary care practice and notably in Family Medicine Groups (FMG).

FMG is the main model for primary care health services in Quebec. An FMG is a group of physicians working closely with other healthcare professionals in the delivery of services to enrolled patients [37]. FMGs operate as private practices for physicians but receive additional funding from the Ministry of Health and Social Services to hire clinical nurses and administrative staff. Since 2015, the FMG model has integrated a wider variety of healthcare services, thus promoting collaboration between a variety of healthcare professionals as well as providing coordinated healthcare to patients. FMGs now at a minimum include physicians, nurse practitioners, clinical nurses, social workers, and pharmacists. Based on their patient population and resources, FMGs can also expand the services offered and hire, for example, psychologists, nutritionists, physiotherapists or occupational therapists. While most FMGs act as independent clinics, some of them are affiliated with universities and incorporate training for residents and interns. These are called University Family Medicine Groups (UFMG).

### Objectives

The general objective of this study is to support primary care teams to improve current AA practice (including physicians, nurse practitioners and administrative staff) and to implement AA for the rest of the care team. We intend to use an external facilitation approach to promote interprofessional collaboration, improve and streamline AA processes for physicians and nurse practitioners, and help healthcare professionals who have not been practicing AA to implement the model.

The specific objectives are to monitor and evaluate organizational change by focusing on different aspects of the facilitation and improvement process:

1. Describe the processes of an externally-facilitated QI intervention on the implementation of AA with primary care teams;
2. Document the various strategies implemented by PHC team to improve AA and measure their impact;
3. Analyze the experience of the introduction of the AA model for social workers, pharmacists, clinical nurses and other PHC professionals.

## Methods/design

### Intervention

For this study, we will support teams in four UFMG and one FMG in the implementation of AA in their clinics, while promoting a quality improvement culture and implementing practice facilitation activities over a period of 18 months.

The five clinics have been selected using purposeful sampling [38, 39], with the objective of covering a wide range of organizational structures and contexts, including geographical and socioeconomical differences. Perceived differences in the implementation level of AA have also been a determining factor. Participating clinics include 5 to 22 family physicians and 1 to 14 nurses each, and all of them include at least one social worker and one pharmacist. The smaller clinics do not provide other services, but the larger ones include services such as psychology, physiotherapy, nutrition and respiratory therapy. Each participating clinic is also represented by a patient who will collaborate in the research process (data collection, analyses and interpretation) as well as the QI intervention, and will be involved in the dissemination process.

The QI intervention lies on two key activities within each clinic: interprofessional reflective sessions and PDSA cycles.

### Interprofessional reflective sessions

An essential condition for people to become involved in organizational change is that they arrive at a shared understanding of the issues to be solved or the challenges to be overcome, which requires a strong commitment from the clinics and their teams [12, 25, 40, 41]. To this end, we will organize clinic-wide meetings labeled *reflective sessions*. These sessions will occur in each clinic approximately every three months and involve all team members, including clinical, administrative and management teams.

During these sessions, we will identify and prioritize AA improvement aims within each local context. First, a series of AA-related indicators, populated with the clinic’s electronic medical health record data, will be presented to the group (see Table 1). Through customized facilitation activities, participants will be asked to generate and prioritize ideas to improve AA while accounting for concerns of all team members. These activities will aim to maximize engagement of the team members, and to foster discussions and collaboration among them. Examples of the facilitated activities are brainstorming sessions, design thinking sessions, or any other activities that might address the specific AA-related issues faced by the team [49]. The end result of the session includes: an improvement aim; identifying the goal(s) the team would like to achieve before the next reflective session; and identifying who will benefit from this improvement and how change will be measured.

**Table 1:** Data collection schedule

Variables	Schedule			Data source
AA variables				
3rd next appointment delay % of appointments available within 48 h Number of missed appointments Continuity of care Interprofessional integration	Collected on a weekly basis			Electronic medical records
Patient variables				
	Baseline	9 months	18 months	
Patient experience AA knowledge	X		X	Patient questionnaire [42–46]
Team variables				
Willingness to change	X		X	Commitment to change questionnaire [47]
Interprofessional collaboration	X	X	X	AITCS-II questionnaire [48]

### PDSA Cycles

Following a first reflective session, we will implement a local governance committee at each of the 5 clinics, comprised of at least one team member from all categories of staff (e.g., physicians, nurses, social workers, administrative staff).

These committees will have the responsibility to implement the aims decided during the reflective session through monthly or more frequent PDSA cycles. Supported by the research team that act as external facilitators, they will convene to review the clinic’s aims, develop (plan) and implement changes (do), monitor the effect of these changes on key indicators (study), and then review the process in order to maintain changes or adjust future actions (act) [15, 17, 18]. These meetings will provide regular monitoring of each team’s progress, and will help to facilitate improvement.

Once a cycle is complete (approximately three months following each interprofessional reflective session), we will reconvene all team members for another reflective session. The local governance committee will present the changes implemented during the last three months. The objective of these additional sessions will be to review the changes, address new issues that might have come up, and decide whether to build upon the changes that have been implemented within the three-month period and/or add new QI aims. This also provides a regular meeting-point for all team members to voice their concerns and ideas, which can then be used by the local governance committee during the PDSA cycles.

### Study design

#### Objective 1: Describe the processes of an externally-facilitated QI intervention on the implementation of AA with primary care teams

We will use a longitudinal qualitative component consisting of interviews with multiple stakeholders from each of the clinics [50]. A first wave of interviews will be conducted with selected team members 6 months following the launch of the project and a second wave will be conducted at 18 months, towards the end of the intervention.

The interviews will be aimed at physicians, nurse practitioners and administrative staff. We expect to recruit between 20 and 30 participants, distributed across the five clinics involved [51]. Maximum variation with respect to profession and experience with AA will be ensured when recruiting participants [39]. Interviews will cover the context of the intervention, the facilitation techniques used, and the overall support and structure provided by the research team. This will allow for the identification of factors that helped or hindered the improvement of the AA model using a QI approach.

The interviews will be analyzed through an inductive process of thematic analysis by a research assistant, using the NVivo software [52]. The first interviews will be transcribed and coded using the initial coding grid. Then, we will identify missing themes or areas of interest from the initial interviews in order to supplement both the interview questions and the coding grid. Multiple waves of interviews will be scheduled with health professionals until we identify that the data has been saturated.

In addition to the qualitative component, individual AA implementation levels will be documented from clinicians and team members at each site, using self-evaluations questionnaires inspired by similar tools [53–57] which will be collected at baseline, 9 and 18 months. In addition, we will monitor two variables over the course of the study in order to measure willingness to change and interprofessional collaboration within each team. Willingness to change and engagement towards AA implementation will be assessed using a modified version of the “Commitment to change” questionnaire, [47] while the interprofessional collaboration level will be assessed using the Assessment of Interprofessional Team Collaboration Scale II [48]. These questionnaires will be administered to all team members at baseline, 9 and 18 months. We will then analyze the changes in these variables over time using mixed linear regression models. This data will also be cross-referenced with the qualitative data extracted from the qualitative interviews, either to suggest new analysis of the quantitative data or to help build the qualitative coding grid.

#### Objective 2: Document the various strategies implemented by PHC team to improve AA and measure their impact

This objective entails two related activities, first to document then measure impacts of improved AA [58, 59].

We will first map out the various aims and the related implementation strategies used by the local governance committees to improve or introduce AA. This will provide a rich portrait of what can be done to facilitate AA-related changes in primary care. Electronic medical records will then be used to measure the impact of the various aims on AA (Table 1). For example, the third next appointment delay and the number of missed appointments are two common indicators to measure an AA practice [1].

Five indicators will be measured regularly:

1. The **third next appointment** is an indicator of delays in obtaining a medical appointment, and is calculated by counting the number of days before the third next available appointment in any professional’s schedule. This helps to identify the level of access for patients, and can be addressed by eliminating some of the short-term variations in health care professional’s schedule [60].
2. The **percentage of appointments available in 48 h** is calculated by dividing the number of available appointments in the next 48 h by the total number of appointment slots in this same period. This indicates the likelihood that a patient with urgent care needs can be seen by his physician in a timely manner.
3. **Missed appointments** refers to the number of appointments missed by patients; higher levels of access for patients have been correlated with a lower rate of missed appointments.
4. **Continuity of care** is measured as whether or not the patient is seen by their own family physician or by another physician, resident or nurse practitioner from the team; and.
5. **Interprofessional integration** is measured as whether the patient is taken care of by a physician or by a specialized health professional, such as a social worker or clinical nurse. This indicator addresses interprofessional collaboration within AA.

These indicators will be collected by a research assistant from the electronic medical records of each clinic on a weekly or monthly basis (depending on the outcome).

Descriptive statistical analysis of these variables assessing changes in each clinic will be cross-referenced with the strategies used using run charts [58] and control charts [59]. The follow-up of each indicator for all clinicians involved will provide sufficient data to draw meaningful conclusions on improvement [61].

We will also assess **patient experience** at each clinic using a customized patient questionnaire based on similar questionnaires used in comparable studies [42–46], local teams’ experiences and recommendations from the patients’ committee. The questionnaire will cover various dimensions of patient experience such as the appointment process, access, communication, team collaboration and knowledge of the AA model. This questionnaire will be distributed in the waiting areas of each clinic at baseline, 9 and 18 months. A convenience sample of 100 patients will be assembled at each collection time point, for each clinic [1]. Within each clinic, such sample size will provide sufficient power (> 80 %) to detect a relatively small Cohen’s effect size (> 0.30).

Further analysis using linear mixed regression models will be carried out in order to measure the impact of practice facilitation on the implementation of AA in primary care clinics, using selected AA indicators, with willingness to change (Commitment to change questionnaire [47]), and interprofessional collaboration (AITCS-II questionnaire [48]) as the outcomes (Table 1). We will control for confounding variables (e.g., team size and composition, number of patients, FMG or UFMG status) in order to evaluate the effectiveness of the longitudinal intervention in different settings.

#### Objective 3: Analyze the experience of the introduction of the AA model for social workers, pharmacists, clinical nurses and other PHC professionals

A qualitative component will be used to explore the implementation of AA in healthcare professionals who had not traditionally worked with the AA model intervention before this study, notably social workers, clinical nurses, and pharmacists. This will take the form of one-on-one semi-structured interviews, focusing on the process of adopting the AA model, the adaptation needed to fit with their own professional guidelines, and the integration of their services in the interprofessional primary care teams. We expect to recruit between 20 and 30 professionals for this objective, or until we perceive a saturation in data collection [22, 62].

Analysis of this qualitative component will be done by a research assistant using NVivo. An initial coding grid will be developed similar to the one used for Objective 1, based on the main principles of AA [1] and on Roger’s attributes of innovations [63], and then categorized using organizational, professional and individual factors. Transversal categories will also be used to identify *barriers* and *facilitators* to AA, and *strategies* used by the professionals in their daily practice. We will follow the same inductive process described in Objective 1, where the initial coding grid will be built upon and modified as needed throughout the analysis process.

### Patient involvement

Our experience as an AA research team and the literature shows that although AA was created to improve patients’ timely access to care and services necessary for their health, efforts are futile if these stakeholders do not benefit from this system [64, 65]. This research aims to work with all stakeholders, including patients to extend this timely appointment system to all FMG professionals and to develop effective materials by and for patients, so that this organizational change is sustainable and highly functional.

The Patient-Partner Initiative (PPI) at the Université de Sherbrooke—supported by the Quebec Strategy for Patient-Oriented Research Support Unit and the Faculty of Medicine and Health Sciences provided expertise to initiate, foster, monitor, and assess high-quality patient-partner relationships consistent with nationally recognized quality criteria. We have recruited five patients from all involved clinics to form a patient’s committee, which is included in all phases of the study. These patients have been selected on a voluntary basis. Two patients from the patients’ committee will be present at each improvement loop meeting, happening every three months, and at the monthly local governance committee meetings as well if possible. They will be able to share the patients’ point-of-view on the discussed subjects and help design objectives and processes while always keeping the patients’ interests in mind. Committee members will also help to design the questionnaires intended for patients, in terms of both its content and form. Finally, they will help develop promotional and educational material as needed, in order to improve patients’ understanding of AA and of the changes implemented during the course of the study.

## Discussion

Improving access to primary care services is one of Quebec’s MSSS top priorities. Support for the implementation of AA is one of the keys to achieving this objective, while improving interprofessional collaboration within the clinic teams. We foresee that the teams recruited will develop better knowledge of each other’s roles and more efficient communication strategies through the study process. Improving the implementation of AA has the potential to improve timely access to a professional who already knows the patient when a problem arises. Also, we expect that improving interprofessional collaboration will lead to better services related to care of complex patients, which has the potential to improve patient outcomes.

This study is among the first aiming to study factors for successful quality improvement initiatives within primary care services. The five chosen clinics represent important variety in size and composition, making it possible to test several strategies for facilitation and to identify key improvements over a long period of time. Moreover, while the quantitative component of this study will identify successful strategies and main challenges to implementation within an interprofessional setting, the qualitative component will provide an in-depth understanding of the team dynamics involved.

One of the challenges of this study is that it requires high engagement of the recruited teams for the improvement process to be successful. Engagement of healthcare professionals in a research and longitudinal improvement project could be laborious. We intend to work very closely with the teams to understand their needs and concerns as they come up, and to maximize their involvement.

The impact will not be limited to the clinics selected for the project. We intend to scale up the results of this study to enable further changes in additional clinics within the healthcare network, at the regional and provincial levels. Data regarding the impact of specific changes made by the local teams can be used to promote better practices among similar clinics, while qualitative data can help to identify key aspects among participating team that might be similar in other clinics. This has the potential to further improve timely access for patients in a variety of services.

Working closely with patients will allow us to fill a major gap in previous AA implementation projects, namely, to adapt AA to better meet patients’ needs and expectations. Also, the creation of explanatory materials for and by patients will allow us to make patients more familiar and autonomous with AA, to make its use more effective and satisfying.

Finally, from a methodological standpoint, this study will provide insights on how a QI project could co-exist with and feed an implementation intervention. As recommended by most tenants of quality improvement [14, 18, 19], facilitation will enable stakeholders to become involved in the change process through reflective practice in order to adapt the changes to their context, making the changes more acceptable and sustainable. The development of the proposed facilitation approach will allow tangible improvements in the implementation of AA, improvement of AA for the professionals already working according to these principles and may eventually be adapted to improve other aspects of primary healthcare delivery.

## Supplementary Information

**Additional file 1:**

## Data Availability

The data that will support the findings of this study are available from the clinics involved, but restrictions apply to the availability of these data, which will be used under license for the current study, and so are not publicly available. Data will however be available from the authors upon reasonable request and with permission of the clinics and other stakeholders, such as Quebec’s Ministry of Health and Social Services.

## Abbreviations

**AA**: Advanced Access.
**PDSA**: Plan-do-study-act.
**FMG**: Family Medicine Group.
**UFMG**: University Family Medicine Group.
**PHC**: Primary Healthcare.
QI: Quality Improvement.
**PPI**: Patient-partner Initiative.
**MSSS**: Ministère de la santé et des services sociaux.
SRAP: Soutien à la recherche axée sur le patient.

## References

[1] Murray M, Berwick DM (2003). Advanced access: reducing waiting and delays in primary care. JAMA.

[2] Bundy DG, Randolph GD, Murray M, Anderson J, Margolis PA (2005). Open access in primary care: results of a North Carolina pilot project. Pediatrics.

[3] Rose KD, Ross JS, Horwitz LI (2011). Advanced access scheduling outcomes: a systematic review. Arch Intern Med.

[4] Fournier J, Heale R, Rietze LL (2012). I can’t wait: advanced access decreases wait times in primary healthcare. Healthcare quarterly (Toronto Ont).

[5] Hudec JC, MacDougall S, Rankin E (2010). Advanced access appointments: effects on family physician satisfaction, physicians’ office income, and emergency department use. Can Fam Physician.

[6] Rivas J (2020). Advanced access scheduling in primary care: a synthesis of evidence. J Healthc Manag.

[7] Breton M, Maillet L, Paré I, Abou Malham S, Touati N. Perceptions of the first family physicians to adopt advanced access in the province of Quebec, Canada. The International Journal of Health Planning and Management. 2016. 10.1002/hpm.2380 27605412

[8] Abou Malham S, Touati N, Maillet L, Breton M (2018). The challenges of implementing advanced access for residents in family medicine in Quebec. Do promising strategies exist?. Medical education online.

[9] Abou Malham S, Touati N, Maillet L, Gaboury I, Loignon C, Breton M (2017). What Are the Factors Influencing Implementation of Advanced Access in Family Medicine Units? A Cross-Case Comparison of Four Early Adopters in Quebec. International journal of family medicine.

[10] Chapman J, Zechel A, Carter Y, Abbot S (2004). Systematic review of recent innovations in service provision to improve access to primary care. Br J Gen Pract.

[11] Deming WE. The New Economics of Industry and Government. GWU Continuing Engineering Education Program; 1989.

[12] Langley GJ, Moen RD, Nolan KM, Nolan TW, Norman CL, Provost LP. The improvement guide: a practical approach to enhancing organizational performance. John Wiley & Sons; 2009.

[13] Batalden PB, Davidoff F. What is “quality improvement” and how can it transform healthcare? BMJ Publishing Group Ltd; 2007. 10.1136/qshc.2006.022046 PMC2464920 17301192

[14] Scoville R, Little K (2014). Comparing Lean and Quality Improvement.

[15] Knox L, Branch C (2013). The Practice Facilitation Handbook: Training Modules for New Facilitators and Their Trainers.

[16] Institute for Healthcare Improvement. Science of Improvement: How to Improve 2018 [Available from: http://www.ihi.org/resources/Pages/HowtoImprove/ScienceofImprovementHowtoImprove.aspx.

[17] Lemire N, Leviak E (2011). L’amélioration en santé: diriger, réaliser, diffuser.

[18] Bowen HK, Spear SJHBR. Decoding the DNA of the Toyota production system. 1999;99.

[19] Linderman K, Schroeder RG, Zaheer S, Choo AS (2003). Six Sigma: a goal-theoretic perspective. J Oper Manag.

[20] Knudsen SV, Laursen HVB, Johnsen SP, Bartels PD, Ehlers LH, Mainz, JJBhsr. Can quality improvement improve the quality of care? A systematic review of reported effects and methodological rigor in plan-do-study-act projects. 2019;19(1):1–10. 10.1186/s12913-019-4482-6 PMC6778385 31585540

[21] Wells S, Tamir O, Gray J, Naidoo D, Bekhit M, Goldmann DJBq, et al. Are quality improvement collaboratives effective? A systematic review. 2018;27(3):226–40. 10.1136/bmjqs-2017-006926 29055899

[22] Kringos DS, Sunol R, Wagner C, Mannion R, Michel P, Klazinga NS (2015). The influence of context on the effectiveness of hospital quality improvement strategies: a review of systematic reviews. BMC Health Serv Res.

[23] Kringos DS, Sunol R, Wagner C, Mannion R, Michel P, Klazinga NS, et al. The influence of context on the effectiveness of hospital quality improvement strategies: a review of systematic reviews. BMC Health Serv Res 2015;15(1):1-13. 10.1186/s12913-015-0906-0 PMC4508989 26199147

[24] Backhouse A, Ogunlayi F. Quality improvement into practice. BMJ. 2020;368. 10.1136/bmj.m865 PMC7190269 32234777

[25] Baskerville NB, Liddy C, Hogg W (2012). Systematic review and meta-analysis of practice facilitation within primary care settings. Annals of Family Medicine.

[26] Liddy C, Laferriere D, Baskerville B, Dahrouge S, Knox L, Hogg W (2013). An overview of practice facilitation programs in Canada: current perspectives and future directions. Healthcare policy = Politiques de sante.

[27] Alagoz E, Chih M-Y, Hitchcock M, Brown R, Quanbeck A (2018). The use of external change agents to promote quality improvement and organizational change in healthcare organizations: a systematic review. BMC Health Serv Res.

[28] Mader EM, Fox CH, Epling JW, Noronha GJ, Swanger CM, Wisniewski AM (2016). A Practice Facilitation and Academic Detailing Intervention Can Improve Cancer Screening Rates in Primary Care Safety Net Clinics. Journal of the American Board of Family Medicine: JABFM.

[29] Russell G, Lane R, Parker S, Litt J, Mazza D, Lloyd J, et al. Preventive Evidence into Practice: what factors matter in a facilitation intervention to prevent vascular disease in family practice? BMC Family Practice. 2019;20(1):N.PAG-N.PAG. 10.1186/s12875-019-0995-7 PMC6688202 31395020

[30] Kaplan HC, Brady PW, Dritz MC, Hooper DK, Linam WM, Froehle CM, et al. The influence of context on quality improvement success in health care: a systematic review of the literature. 2010;88(4):500–59. 10.1111/j.1468-0009.2010.00611.x PMC3037175 21166868

[31] Miller R, Weir C, Gulati S. Transforming primary care: scoping review of research and practice. Journal of Integrated care. 2018. 10.1108/JICA-03-2018-0023 PMC6091658 30166943

[32] Ritchie MJ, Parker LE, Edlund CN, Kirchner JE (2017). Using implementation facilitation to foster clinical practice quality and adherence to evidence in challenged settings: a qualitative study. BMC Health Services Research.

[33] Ye J, Zhang R, Bannon JE, Wang AA, Walunas TL, Kho AN, et al. Identifying practice facilitation delays and barriers in primary care quality improvement. 2020;33(5):655–64. 10.3122/jabfm.2020.05.200058 32989060

[34] Parchman ML, Anderson ML, Coleman K, Michaels LA, Schuttner L, Conway C (2019). Assessing quality improvement capacity in primary care practices.

[35] Laberge M, Gaudreault M (2019). Promoting access to family medicine in Québec, Canada: Analysis of bill 20, enacted in November 2015. Health Policy.

[36] Schoen C, Osborn R, Squires D, Doty MM (2013). Access, affordability, and insurance complexity are often worse in the United States compared to ten other countries. Health Aff.

[37] Breton M, Lévesque J-F, Pineault R, Hogg W (2011). Primary Care Reform: Can Quebec’s Family Medicine Group Model Benefit from the Experience of Ontario’s. Family Health Teams? Healthcare policy = Politiques de sante.

[38] Palinkas LA, Horwitz SM, Green CA, Wisdom JP, Duan N, Hoagwood K. Purposeful Sampling for Qualitative Data Collection and Analysis in Mixed Method Implementation Research. Administration Policy in Mental Health Mental Health Services Research. 2015;42(5):533–44. 10.1007/s10488-013-0528-y PMC4012002 24193818

[39] Patton MQ. Qualitative research. Encyclopedia of statistics in behavioral science. 2005.

[40] Crabtree BF, Nutting PA, Miller WL, McDaniel RR, Stange KC, Jaen CR, et al. Primary care practice transformation is hard work: insights from a 15-year developmental program of research. 2011;49(Suppl):S28. 10.1097/MLR.0b013e3181cad65c PMC3043156 20856145

[41] Silver SA, Harel Z, McQuillan R, Weizman AV, Thomas A, Chertow GM, et al. How to begin a quality improvement project. 2016;11(5):893–900. 10.2215/CJN.11491015 PMC4858490 27016497

[42] Haggerty JL, Roberge D, Freeman GK, Beaulieu C, Bréton M (2012). Validation of a generic measure of continuity of care: when patients encounter several clinicians. Ann Fam Med.

[43] Campbell J, Smith P, Nissen S, Bower P, Elliott M, Roland M (2009). The GP Patient Survey for use in primary care in the National Health Service in the UK – development and psychometric characteristics. BMC Family Practice.

[44] Haggerty JL, Levesque J-F (2017). Validation of a new measure of availability and accommodation of health care that is valid for rural and urban contexts. Health Expect.

[45] Safran DG, Kosinski M, Tarlov AR, Rogers WH, Taira DA, Lieberman N, et al. The Primary Care Assessment Survey: tests of data quality and measurement performance. 1998:728 – 39. 10.1097/00005650-199805000-00012 9596063

[46] Henry BW, Rooney DM, Eller S, Vozenilek JA. McCarthy DMJPe, counseling. Testing of the Patients’ Insights and Views of Teamwork (PIVOT) Survey: A validity study. 2014;96(3):346–51. 10.1016/j.pec.2014.06.002 24976630

[47] Strating MM, Nieboer AP (2013). Explaining variation in perceived team effectiveness: results from eleven quality improvement collaboratives. J Clin Nurs.

[48] Orchard C, Pederson L, Read E, Mahler C, Laschinger H (2018). Assessment of Interprofessional Team Collaboration Scale (AITCS): Further Testing and Instrument Revision. Journal of Continuing Education in the Health Professions.

[49] Lipmanowicz H, McCandless K. The surprising power of liberating structures: Simple rules to unleash a culture of innovation. Liberating Structures Press Seattle, WA; 2013.

[50] Derrington ML. Qualitative longitudinal methods: Researching implementation and change. Sage Publications; 2018.

[51] Saunders MN, Townsend K (2016). Reporting and justifying the number of interview participants in organization and workplace research. Br J Manag.

[52] Miles MB, Huberman AM, Saldaña J. Qualitative data analysis: A methods sourcebook. Sage publications; 2018.

[53] Health Quality Ontario. Advanced access and efficiency workbook for primary care. Toronto: Queen’s printer for Ontario; 2012.

[54] Lukas C, Meterko M, Mohr D, Seibert MN (2004). The implementation and effectiveness of advanced clinic access.

[55] Fleuren MA, Paulussen TG, Van Dommelen P, Van Buuren S (2014). Towards a measurement instrument for determinants of innovations. Int J Qual Health Care.

[56] Health Quality Ontario. Advanced Access and Efficiency Workbook for Primary Care Toronto: Queen’s Printer for Ontario; 2012 [Available from: www.hqontario.ca/Portals/0/…/qi-aae-interactive-workbook-en.pdf.

[57] Institut canadien d’information sur la santé. Sondages dans les cliniques de soins de santé primaires 2013 [Available from: https://www.cihi.ca/sites/default/files/document/info_phc_handout_fr.pdf.

[58] Lloyd RC. Understanding variation with Run Charts. In: Learning JaB, editor. Quality Health Care: A guide to developing and using indicators. 2d ed. Burlington2019. p. 187–209.

[59] Lloyd RC. Understanding variation with Shewhart charts. In: Learning JaB, editor. Quality Health Care: A guide to developing and using indicators. 2d ed. Burlington2019. p. 210 – 58.

[60] Pascucci D, Sassano M, Nurchis MC, Cicconi M, Acampora A, Park D, et al. Impact of interprofessional collaboration on chronic disease management: findings from a systematic review of clinical trial and meta-analysis. Health Policy. 2020. 10.1016/j.healthpol.2020.12.006 33388157

[61] Lloyd R. Quality Health Care: A Guide to Developing and Using Indicators. Jones & Bartlett Learning; 2017.

[62] Saunders B, Sim J, Kingstone T, Baker S, Waterfield J, Bartlam B (2018). Saturation in qualitative research: exploring its conceptualization and operationalization. Quality quantity.

[63] Rogers EM. Diffusion of innovations. Simon and Schuster; 2010.

[64] Dixon S, Sampson FC, O’Cathain A, Pickin M (2005). Advanced access: more than just GP waiting times?. Fam Pract.

[65] Salisbury C, Goodall S, Montgomery AA, Pickin DM, Edwards S, Sampson F (2007). Does Advanced Access improve access to primary health care?. Questionnaire survey of patients.

