# Supplementary material for: Interprofessional advanced access – a quality improvement protocol for expanding access to primary care services
Source: BMC Health Serv Res. 2021 Aug 13;21:812. doi: 10.1186/s12913-021-06839-w (PMC8361639; doi:10.1186/s12913-021-06839-w)
Supplement: Supplementary file 1 — Additional file 1: [file 12913_2021_6839_MOESM1_ESM.pdf]

Assessment of Interprofessional Team Collaboration Scale II (AITCS-II)
© C Orchard, 2015

The AITCS is a diagnostic instrument that is designed to measure the interprofessional collaboration among team members. It consists of 23 statements considered characteristic of interprofessional collaboration (how team works and acts). Scale items represent three elements that are considered to be key to collaborative practice. These subscales are: (1) Partnership— 8 items, (2) Cooperation—8 items, and (3) Coordination—7 items.

Scoring AITCS
Respondents indicate their general level of agreement with items on a 5-point rating scale that ranges from 1 = "Never"; 2 = "Rarely"; 3 = "Occasionally"; 4 = "Most of the time"; to 5 = "Always". These ratings produce scores from 23 to 115. It takes approximately 10 minutes to complete.

Demographic Information

Please enter the last four digits of your employee ID number in these boxes: □□□□

Please check □ the category you belong to:

Gender: □ Male □ Female **Age:** ____years

Employment Status: □ FT □ PT □ Casual

Educational Preparation
□ Certificate
□ Diploma
□ Other (specify): __________
□ Bachelor Degree
□ Masters Degree

Please check one of the following discipline categories:
□ Audiologist
□ Clinical Kinesiologist
□ Clinical Psychologist
□ Dental Assistant
□ Dentist
□ Dietary Aid
□ Dietitian (Nutritionist)
□ Imaging Technologist
□ Laboratory Technologist
□ Nursing: Registered Nurse
□ Nursing: Practical Nurse
□ Occupational Therapist
□ Physical Therapist (Physiotherapist)
□ Pharmacy
□ Paramedics
□ Physician (Medicine)
□ Personal Support Worker
□ Speech Language Pathologist
□ Social Worker
□ Spiritual/Pastoral Care
□ Recreational Therapist
□ Respiratory Therapist
□ Therapy Assistant
□ Other (please specify) __________

Please indicate:
Years in practice (*since achieving license to practice*):__________; Years with your current team: __________

Assessment of Interprofessional Team Collaboration Scale

Instructions:

Note: Several terms are used for the person who is the recipient of health and social services. For the purpose of this assessment, the term 'patient' will be used. While acknowledging other terms such as 'client' 'consumer' and 'service user' are preferred in some disciplines/jurisdictions.

Please ***circle the value*** *which best reflects how you currently feel your team and you, as a member of the team, work or act within the team.*

1	2	3	4	5
Never	Rarely	Occasionally	Most of the time	Always

Section 1: PARTNERSHIP

When we are working as a **team**[1] all of my team members…..

1	include patients in setting goals for their care	1 2 3 4 5
2	listen to the wishes of their patients when determining the process of care chosen by the team	1 2 3 4 5
3.	meet and discuss patient care on a regular basis	1 2 3 4 5
4.	coordinate health and social services (e.g. financial, occupation, housing, connections with community, spiritual) based upon patient care needs	1 2 3 4 5
5.	Use consistent communication with to discuss patient care	1 2 3 4 5
6.	Are involved in goal setting for each patient	1 2 3 4 5
7.	encourage each other and patients and their families to use the knowledge and skills that each of us can bring in developing plans of care	1 2 3 4 5
8.	work with the patient and his/her relatives in adjusting care plans	1 2 3 4 5

[1] A team can be defined as any interactions between one or more health professionals on a regular basis for the purposes of providing patient care.

Section 2: COOPERATION

When we are working as a **team** all of my team members…..

9.	share power with each other	1	2	3	4	5
10.	respect and trust each other	1	2	3	4	5
11.	are open and honest with each other	1	2	3	4	5
12.	make changes to their team functioning based on reflective reviews	1	2	3	4	5
13.	strive to achieve mutually satisfying resolution for differences of opinions	1	2	3	4	5
14.	understand the boundaries of what each other can do	1	2	3	4	5
15.	understand that there are shared knowledge and skills between health providers on the team	1	2	3	4	5
16.	establish a sense of trust among the team members	1	2	3	4	5

Section 3: COORDINATION

When we are working as a **team** all of my team members…..

17.	apply a unique definition of ***Interprofessional collaborative practice*** to the practice setting	1	2	3	4	5
18.	equally divide agreed upon goals amongst the team	1	2	3	4	5
19.	encourage and support open communication, including the patients and their relatives during team meetings	1	2	3	4	5
20.	use an agreed upon process to resolve conflicts	1	2	3	4	5
21.	support the leader for the team varying depending on the needs of our patients	1	2	3	4	5
22.	together select the leader for our team	1	2	3	4	5
23.	openly support inclusion of the patient in our team meetings	1	2	3	4	5

Revised version November 16, 2015

Thank you for completion of this questionnaire!

© C Orchard, 2015
